# *In situ* effect of CPP-ACP chewing gum upon erosive enamel loss

**DOI:** 10.1590/1678-7757-2016-0304

**Published:** 2017

**Authors:** Catarina Ribeiro Barros de ALENCAR, Gabriela Cristina de OLIVEIRA, Ana Carolina MAGALHÃES, Marília Afonso Rabelo BUZALAF, Maria Aparecida de Andrade Moreira MACHADO, Heitor Marques HONÓRIO, Daniela RIOS

**Affiliations:** 1 Universidade Estadual da Paraíba, Departamento de Odontologia, Araruna, PB, Brasil; 2 Universidade de São Paulo, Faculdade de Odontologia de Bauru, Departamento de Odontopediatria, Ortodontia e Saúde Coletiva, Bauru, SP, Brasil; 3 Universidade de São Paulo, Faculdade de Odontologia de Bauru, Departamento de Ciências Biológicas, Bauru, SP, Brasil

**Keywords:** Tooth erosion, Dental enamel, Tooth wear, Chewing gum, Casein phosphopeptide-amorphous calcium phosphate

## Abstract

Casein phosphopeptide-amorphous calcium phosphate (CPP-ACP) is able to increase salivary calcium and phosphate levels at an acidic pH. Previous studies demonstrated that a CPP-ACP chewing gum was able to enhance the re-hardening of erosion lesions, but could not diminish enamel hardness loss. Therefore, there is no consensus regarding the effectiveness of CPP-ACP on dental erosion.

**Objective:**

This *in situ* study investigated the ability of a CPP-ACP chewing gum in preventing erosive enamel loss. Material and Methods: During three experimental crossover phases (one phase *per* group) of seven days each, eight volunteers wore palatal devices with human enamel blocks. The groups were: GI – Sugar free chewing gum with CPP-ACP; GII – Conventional sugar free chewing gum; and GIII – No chewing gum (control). Erosive challenge was extraorally performed by immersion of the enamel blocks in cola drink (5 min, 4x/day). After each challenge, in groups CPP and No CPP, volunteers chewed one unit of the corresponding chewing gum for 30 minutes. Quantitative analysis of enamel loss was performed by profilometry (µm). Data were analyzed by Repeated-Measures ANOVA and Tukey’s test (p<0.05).

**Results:**

The use of chewing gum (CPP and No CPP) resulted in lower erosive enamel loss compared with the control group (p<0.05). CPP-ACP chewing gum (CPP) did not improve the protection against erosive enamel loss compared with conventional chewing gum (No CPP) (p>0.05).

**Conclusion:**

The CPP-ACP chewing gum was not able to enhance the anti-erosive effect of conventional chewing gum against enamel loss.

## Introduction

Tooth wear is a progressive and multifactorial process in which erosion, attrition, and abrasion may synergistically act^25^. Although there is no consensus on the diagnostic criteria that differentiates these lesions it has been considered that erosion is the largest contributing factor to the tooth wear in childhood and adolescence^4^. The term erosive tooth wear refers to chemical-mechanical processes in which abrasive forces removes the softened layer attacked by nonbacterial acids, causing tooth hard substance loss^12^. However, tooth hard tissue loss can also occur without significant involvement of abrasion, in cases of prolonged repeated erosive challenges^12^.

Adequate preventive measures will often decrease the erosion progression and reduce the need for immediate restorations^11^. The control of dental erosion requires a combination of strategies that includes the reduction on the frequency of erosive challenges and the enhancement of salivary defenses^12^. The latter can be achieved, among other ways, by the use of chewing gum^22^. In addition, therapies including fluoride applications, in high-concentration, acidic formulations and polyvalent sources like as stannous fluoride have been described to yield positive results in erosive treatment^8^.

Based on CPP-ACP promising results for subsurface carious lesions treatment^9,14^, some studies have been conducted to test this agent against dental erosion when added to pastes or mousses^16,19,30^, sports drinks^18^ or soft drinks^6,13^, and contained in chewing gum^1,10,17^. However, there is no consensus regarding the effectiveness of CPP-ACP on dental erosion and its mechanism of action in relation to erosion is not fully understood^30^.

At an acidic pH, amorphous calcium phosphate (ACP) will separate from casein phosphopeptide (CPP), thereby increasing salivary calcium and phosphate levels. The localized increase of calcium and phosphate degree of saturation will promote carious lesion remineralization by the diffusion of the higher concentration gradient to the lowest^21^. Confirming this effect, in previous studies of erosion, the *in situ* use of chewing gum with CPP-ACP was able to enhance the rehardening of erosion lesions^1,17^. Additionally, the increase of salivary calcium and phosphate levels by CPP-ACP at acidic pH might also inhibit demineralization^20^. Recently, an *in situ* study showed that the use of chewing gum immediately before a short erosive demineralization was able to diminish enamel hardness loss, however, the presence of CPP-ACP in the chewing gum could not enhance this protective effect^10^. It is important to point out that *in situ* models simulating initial erosion can give the first insights regarding preventive measures, but the interplay of erosive challenges and the protective mechanisms of the oral cavity along time are important to confirm the treatment effect. This study was designed to evaluate the effect of CPP-ACP chewing gum, considering the role process of erosion, trying to clarify the impact of remineralization and demineralization on the final degree of enamel loss. Therefore, the aim was to investigate the ability of CPP-ACP chewing gum in preventing enamel loss by erosive challenges. The null hypothesis tested was that there would be no difference between the CPP-ACP chewing gum and a conventional sugar free chewing gum in reducing erosive enamel loss.

## Material and Methods

### Ethical standards

Ethical approval for the study was granted by the local Institutional Ethics Committee (protocol no. 169). This study was conducted in full accordance with the Declaration of Helsinki. Eight healthy adult subjects (six female and two males) with an average age of 27.2 years (range 23-38 years) took part of this study after giving informed consent form. Volunteers fulfilled the inclusion criteria: residing in the same fluoridated area (0.70 mg F/l), physiological stimulated salivary flow rate (>1ml/min), and adequate oral health (with no caries or erosion lesions). They also did not violate the exclusion criteria: systemic illness, pregnancy or breastfeeding, under orthodontic intervention, and use of fluoride compounds in the last two months.

### Experimental design

**Figure 1:**
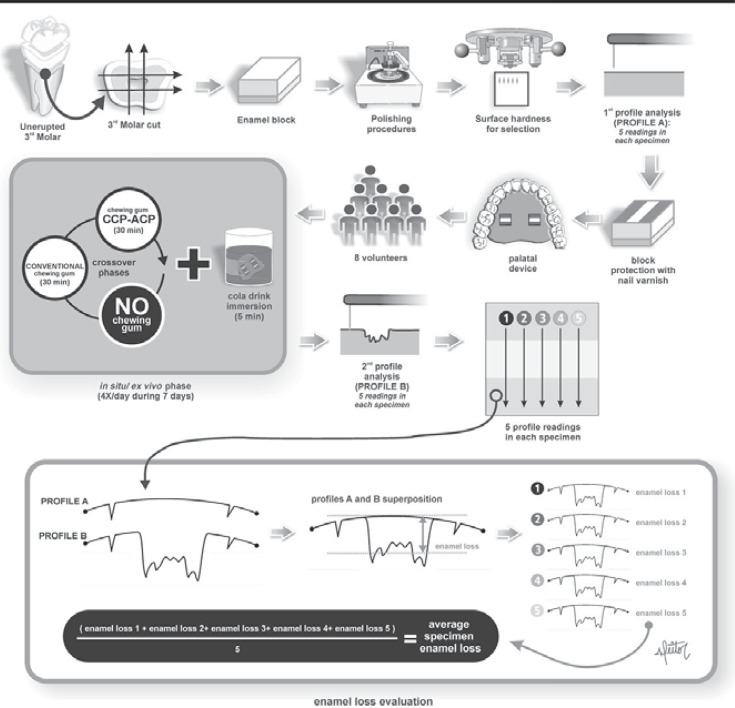
Flowchart of the experimental design

**Figure 2:**
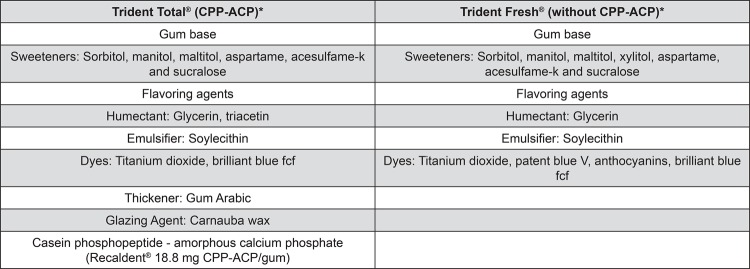
Main components of the chewing gums under study, according to manufactures information

The experimental design is illustrated in Figure 1. The study was conducted with a single-blind and randomized protocol of three-way crossover phases of seven days with an interval of one week between them. The factor under evaluation was treatment at three levels (GI – sugar free chewing gum with 18.8 mg of CPP-ACP; GII – conventional sugar free chewing gum without CPP-ACP; and GIII – negative control group, without chewing gum). Main components of the chewing gums are described in Figure 2. Eight volunteers wore acrylic palatal devices, each containing two human enamel blocks. Four daily erosive cycles (Coca Cola^®^ Ribeirão Preto, Brazil, pH 2.4, 0.32 ppm F) were performed, after which the palatal devices were washed in running water. Thereafter, volunteers replaced the appliance into the mouth and chewed one unit of the chewing gum (Trident Total^®^ or Trident Fresh^®^ - Kraft Foods/Cadbury Adams, Bauru, SP, Brazil for groups CPP and No CPP, respectively) for 30 minutes. Quantitative analysis of enamel loss was performed by profilometry.

*Information given by Trident, Cadbury Adams (Indústria e Comércio, Bauru, São Paulo, Brazil).

### Enamel samples preparation

Enamel blocks (4x4x3 mm) were obtained from unerupted human third molars recently extracted and kept in 0.1% thymol solution at pH 7.0. The blocks were cut using a ISOMET low speed saw cutting machine (Buehler Ltd., Lake Bluff, IL, USA) with two diamond disks (Extec Corp., Enfield, CT, USA), which were separated by a 4 mm thickness spacer. The surfaces of the blocks were ground flat with water-cooled silicon carbide discs (320, 600, and 1200 grade papers; Buehler, Lake Bluff, IL, USA) and polished with felt paper wet by diamond spray (1 µm polishing particle size; Buehler, Ltd., Lake Bluff, IL, USA). Blocks were cleaned using an ultrasonic device for 10 minutes and checked regarding the presence of white spots and cracks using a microscope (x40).

The surface hardness (SH) was determined using the average values of five indentations performed at distances of 100 µm from each other (Knoop diamond, 25 g, 10 s, Hardness tester from Buehler, US). Seventy-two enamel blocks with mean SH of 343.9 (± 15.5 KHN) were selected and sterilized by exposure to ethylene oxide gas.

### First profile evaluation

Enamel blocks were marked with a scalpel blade No. 11 (Embramac, Itapira, SP, Brazil) for standardization of two control areas with width of 1.0 mm (edges) and one test area with 2.0 mm (center). The baseline profile of enamel blocks was evaluated by a contact profilometer (Marh, MarSurf GD 25, Göttingen, Germany) using a contour software (MarSurf XCR 20). Blocks were fixed in a device to standardize their position and to allow the record of the location of each profile. In each block, five readings were made at predetermined distances of 2.25, 2.0, 1.75, 1.5, and 1.25 µm. The graphics (profile) of each read were individually saved. Then, control areas located on the two-thirds in the edges of the blocks were protected with cosmetic nail varnish (Maybelline Colorama, Cosbra Cosmetics Ltda, São Paulo, SP, Brazil) to maintain their integrity during the *in situ* phase.

### Sample size calculation

Sample size calculation was based on a pilot *in situ* study with three volunteers. A sample size of eight volunteers was estimated based on a α-error of 5%, β-error of 20%, 0.9 µm as estimated standard deviation, and 1.51 µm as minimum detectable means difference.

### Volunteers and *in situ* phase

The intraoral palatal devices were made of the upper arch for each volunteer with acrylic resin on the plaster model. Each palatal device had two vertical rows, one on the right and the other on the left side, with one cavity (6x6x3 mm) in each side, for enamel blocks fixation. Samples were fixed with wax and were carefully adapted to the level of the resin surface of the acrylic device, in order to avoid dental plaque accumulation.

Seven days prior to and during the experiment period, the volunteers brushed their teeth with standardized fluoride toothpaste (Total 12, 1.100 ppm F, Colgate, Brazil) three times a day. The volunteers were instructed not to use any other fluoride product.

Intraoral palatal devices were installed at the day before the start of the experiment, in the evening after the last oral hygiene to allow the formation of acquired enamel pellicle. During the following seven days, erosive challenges were extraorally performed four times a day (8 h, 12 h, 16 h, and 20 h). In each challenge, the palatal device was immersed in a cup containing 150 mL of a freshly opened bottle of a cola soft drink (Coca Cola^®^ Ribeirão Preto, Brazil, pH 2.4, 0.32 ppm F) for 5 minutes.

After the erosive challenge, intraoral devices were washed in tap water and reinserted into the oral cavity. Then, for GI (Sugar free chewing gum with CPP-ACP) and GII (Conventional sugar free chewing gum), the volunteers chewed one unit of the respective gum for 30 minutes. After this time, the chewing gum was discarded, and the appliance was worn for at least 2 hours before its removal for another immersion in erosive beverage or for eating.

Volunteers were instructed to use the intraoral palatal device continuously (20 h/day) except during meals (four meals daily of 1 hour each). In the periods of range of use, the appliances were kept in a plastic box, wrapped in gauze moistened in water supply (Bauru, São Paulo, Brazil – 0.7 ppm F).

### Final prolife evaluation and enamel loss measurement

After the *in situ* phase, enamel blocks were removed from intraoral palatal devices. The cosmetic nail varnish was mechanically removed by using the tip of a scalpel blade positioned in the angle between the base and external wall of the block surface. Enamel blocks were repositioned on the table of the profilometer according to its baseline position and five readings were performed as previously described. Baseline and final graphs (profile) of each one of the five readings were superimposed using MarSurf XCR 20 software (Figure 1). The average points were selected for measuring the distance between the graphs in height, in order to define the enamel loss in micrometers.

### Statistical analysis

Statistical analysis was performed with SigmaPlot version 12.3 (2011 Systat Software, Germany). The assumption of equality of variances were satisfied and Shapiro-Wilk test checked the normal distribution of the data (p>0.05). Repeated- Measures ANOVA followed by Tukey’s test were applied. The significant limit was set at 5%.

## Results

All eight volunteers completed the *in situ* protocol and no side effects were reported. Table 1 shows that the chewing gum use (GI and GII) resulted in a significantly lower erosive enamel loss compared with the control group (GIII – without gum). Chewing gum containing CPP-ACP (GI) did not improve the protection against erosive enamel loss in comparison with the conventional chewing gum (GII) (p>0.05).

**Table 1:** Means and standard deviation values of erosive enamel loss (µm) for experimental groups

Experimental Groups	Mean ± SD
GI (CPP-ACP)	5.2 ± 2.8^a^
GII (without CPP-ACP)	3.8 ± 1.5ª
GIII (no chewing gum)	6.8 ± 3.5^b^

Groups whose means are followed by distinct letters differ significantly. (Repeated Measures ANOVA/Tukey’s Test, p<0.05).

## Discussion

The ideal prevention and control of erosive tooth wear involves the removal of causal factors. However, this approach is difficult and not always practiced. For this reason, different therapies have been proposed and studied^2,8,15^.

One of these therapies is the use of CPP-ACP. Promising results were obtained from studies that evaluated the CPP-ACP remineralizing capacity on eroded lesions^1,16,17,19^. On the other hand, other studies have found some limitation in the effect of CPP-ACP when the product was applied before the initial acid attack^10^ or in erosive cycling protocols, in which fluoride was used as the positive control^28^. The knowledge of the effect of chewing gum containing CPP-ACP on erosive lesions is still limited in the literature.

The results of the present study showed that the use of chewing gum diminished the enamel loss from erosive challenges when compared with no chewing gum. This is in accordance with previous findings reporting a reduction in erosive tooth wear with the use of sugar-free chewing gum for 30 minutes after erosive challenges^22,23^. The anti-erosive potential of different agents has been supported by the repair of eroded lesions as a result of mineral precipitation^2^. Based on this, it was hypothesized that the protective effect of the chewing gum was due to mechanical and gustatory salivary stimulation that elevate calcium concentrations in saliva, promoting mineral deposition on the eroded lesion, thereby reducing enamel loss in subsequent acid attack. However, the magnitude of the protection by mineral precipitation can be questioned when considering overall erosion prevention. The histological features of eroded enamel indicate that part of the tissue is etched away during the erosive attack resulting in limited areas for remineralization to take place^25^.

There are two studies that showed an improvement on rehardening (remineralization) of eroded enamel after the use of chewing gum containing CPP-ACP in comparison to the salivary stimulus by regular gum^1,17^. The effect of casein on enamel remineralization has been associated with a subsequent increase in tissue resistance and reduced solubility of tooth structures to acid attack^9^. However, when considering cycles of demineralization and remineralization, as applied in the present study, the previously found rehardening effect of CPP-ACP on eroded enamel did not reflect in higher enamel resistance against erosive enamel loss when compared with the effect promoted by salivary stimulus using a regular gum. Results did not show differences in the erosion-preventive effect between regular and CPP-ACP containing gums. Therefore, there is no evidence that higher mineral precipitation, as promoted by CPP-ACP gums, will provide a less susceptible enamel surface against subsequent erosive challenges.

As suggested by Eisenburger, et al.^5^(2001), *in vitro* remineralization of the eroded surface after 1, 2, and 4 hours in artificial saliva is extremely fragile and susceptible to removal by mechanical forces. The remineralization process would involve the deposition of minerals in zones of enamel porosity rather than the promotion of regeneration of hydroxyapatite crystals in these short periods of remineralization. However, as erosive lesions are vulnerable to other damage immediately after its formation, the goal of remineralization of erosion lesions would be restore mechanical strength as much as possible in order to avoid subsequent losses; though evidence has shown that it is far from being achieved^26^.

When considering erosive enamel loss the most important effect of preventive treatments seems to be surface protection rather than mineral precipitation^8^. The mechanism of enamel protection of CPP-ACP is based on the increase of the degree of saturation of calcium and phosphate when pH drops by erosive challenge, inhibiting demineralization^29^. In addition, it is hypothesized that CPP-ACP ability of forming micelles on the tooth surface^3^ could result in a semi-permeable barrier^24^, which might restrict the reaction of H^+^ ion with the tooth surface and also inhibit the loss of Ca^2^ ions. However, Jordão, et al.^10^ (2016) found that the use of chewing gum immediately before an erosive demineralization can diminish enamel hardness loss and the presence of CPP-ACP in the chewing gum cannot enhance this protective effect. These results were similar to the present study, reinforcing the theses that CPP-ACP is not able to protect enamel against erosion. In contrast, de Oliveira, et al.^15^ (2017), using similar *in situ* protocol, showed that the same commercial CPP-ACP chewing gum resulted in lesser enamel loss compared with regular chewing gum when enamel blocks were subjected to erosion. On the other hand, when erosion was associated to abrasion, the gums showed statistically similar behavior. Therefore, in a more realistic condition, in which erosive enamel wear is enhanced by oral mechanical forces, CPP-ACP shows limited effect. Since in the present study there was no apparatus on the palatal appliance to hinder the forces of the tongue^7,27^, it is speculated that erosion was associated to tongue abrasion and the addition of CPP-ACP was not able to enhance the protection promoted by a conventional chewing gum use, similarly to the results of Oliveira, et al.^15^ (2017).

Based on the results of the present study, the inclusion of CPP-ACP in a chewing gum was not able to enhance the protective effect of the use of a conventional chewing gum against erosive enamel loss. The null hypothesis was accepted. Therefore, to date, there is no evidence that chewing gum containing CPP-ACP should be used to prevent the development or progression of erosive enamel loss.
